# Obesity Epidemic in Brazil and Argentina: A Public Health Concern

**Published:** 2014-06

**Authors:** Alberto K. Arbex, Denise R.T.W. Rocha, Marisa Aizenberg, Maria S. Ciruzzi

**Affiliations:** ^1^Endocrinology Department, Medical Institute for Teaching and Research—IPEMED, Brazil and Federal University of Rio de Janeiro (UFRJ), Brazil; ^2^Law Faculty, University of Buenos Aires (UBA), Brazil

**Keywords:** Jurisprudence, Obesity, Public health, Latin America

## Abstract

The obesity epidemic is rapidly advancing in South America, leading to inevitable health consequences. Argentinian and Brazilian health policies try to become adapted to the new economic and social framework that follows from this epidemic. It is in incipient and ineffective control so far since the prevalence of obesity was not restrained. The Argentine national legislation is more advanced, through the so-called “Ley de Obesidad.” In Brazil, there are numerous local initiatives but still not a comprehensive law. National policies relating to decisions regarding obesity are discussed in this paper. Trends in decisions issued in higher courts of Argentina (Supreme Court of Justice of the Nation—CSJN) and Brazil (Supreme Court of Justice—STF), in the last 15 years, seek to clarify the approach of each country and court's resolutions. Marked differences were found in their positions. Finally, legal and health solutions to this obesity epidemic are proposed.

## INTRODUCTION

Obesity is a worldwide health and sociopolitical problem. Excessive body-weight currently affects over 50% of the Brazilian and Argentinian populations. This picture is similar to other countries in the world nowadays (1,2). Only in 1985, the World Health Organization (WHO) officially recognized obesity as a disease. However, the global prevalence of obesity has doubled since 1980, requiring the attention of authorities (2). The knowledge about this disease has increased since the 1990s. We now have a better, broader understanding of the metabolic and hormonal changes that underlie its development in the human body (3).

### Prevalence of obesity in the Mercosur

The nutrition transition phenomenon has been described in Brazil as in other developing countries (4). Since the 1970s, the occurrence of famine in the population has rapidly decreased but the prevalence of obesity has increased on an even faster rate (5). An Argentinian survey (6) revealed that 50.5% of the population exceeded expected healthy weights. Of these, 34.8% were overweight [a body mass index (BMI) greater than or equal to 25 kg/m^2^] and 14.8% were obese (BMI over 30 kg/m^2^). Regarding gender, 40.3% of females and 59.7% of males were overweight. Among adults, the largest percentage of overweight persons was found in people between 35 and 64 years of age; In Brazil, a study done by the Ministry of Health (7) in 2011 showed that 48.1% of the population was either overweight (33.1%) or obese (15%). In 2006, these figures were 22.7% and 11.4%. Thus, a rise in the incidence of obesity is evident and worrysome (7). A research conducted in 2008-2009 indicated that 50% of men and 48% of women had excess weight, and 12.5% of men and 16.9% of women were obese. Childhood obesity is also a concern. One in every three children between five and nine years of age was overweight. In 34 years, overweight Brazilian adolescents (10 to 19 years) grew from 3.7% to 21.7% in this age-group, indicating a six-fold rise (8).

### Social impact

The public opinion increasingly regards obesity as a growing and dangerous health hazard. The media in both countries actively discuss the national risks of childhood obesity, looking for public and private solutions (9,10). However, no successes have yet been achieved, and the epidemic only increased (6,7). A recent study has indicated that half of the adult population of the United States will be obese by 2030, resulting in a consequent increase in the incidence of diabetes, stroke, and cancer relating to excess weight. Efforts to address the problem are lacking. It has been suggested that a solution can only be found through direct intervention of governments and authorities (11). The implementation of corrective public health policies could be a valid approach towards control of obesity (12). The increasing occurrence of this disease has a significant impact on economic and social structures, swelling both governmental and private healthcare costs. In turn, it generates unprecedented social and juridical demands as the right to health clearly stated in the Federal Constitutions. Thus, the creation and expansion of specific laws that address the issue of obesity are a natural consequence (13). Effective policies to promote healthier weight may bring economic benefits and reduce health costs.

### Legal systems at the Mercosur

Brazil and Argentina share very similar legal systems. Both are representative young democracies (around 30 years old); both have a presidential government system, a still-inadequate income distribution, and belong to the same commonwealth of Mercosur. They share a parliament for the Mercosur and many common laws and health initiatives. They represent, indeed, the two most important economies and countries of South America. In both nations, there are three echelons of litigation: first (entrance level), second, and third (higher) level courts. Issues of national interest involving the country's health policies and constitutional matters can reach the highest level, represented in Brazil by the Supreme Court of Justice (STF) and, in Argentina, by the Supreme Court of Justice of the Nation (CSJN). In this way, judicial decisions can influence government policies towards health (14,15). Equally in Brazil and Argentina, national political power is divided among three branches: the executive, legislative, and judicial ones. Both nations depend on the sharing of power among federal, state and city governments. However, as an exception to so many similar structures, Argentinian federalism is decentralized since its states enjoy more autonomy from the national executive than those of Brazil (15).

Litigation can also be a useful strategy to advancing social rights in developing countries. Juridical decisions may influence public policies, leading to important political and economic consequences (16) as shown by the recent decisions of the superior courts of Brazil and Argentina that affected health. In 2009, the Supreme Court of Brazil allowed the use, under specific conditions, of stem cells for research (17). In 2012, it authorized the abortion of embryos without brains (17). In Argentina, specific legal rules were defined to treat the ‘end of life’ and euthanasia early in 2012 (18). Obesity seems to receive more attention in the courts of Argentina than in those of Brazil as long as specific existing laws touch on this question.

### Public health systems at the Mercosur

In Brazil, the Public Healthcare System (a.k.a. as ‘SUS’) has control over public health. Since the adoption of the last constitution, as of 1988, all former health systems have been merged and placed under its auspices. SUS reaches the entire population but suffers from some obvious limitations, such as a low ‘per-capita’ investment and limited support for 190 million users. In Argentina, three different health systems exist [public health–as in Brazil, the broader covering system, social work (‘obras sociales’), and private practice)], each with different rules. This division of authority makes it more difficult to achieve an ideal management. Therefore, rules that apply to all three systems are often necessary, such as the ‘Ley de Obesidad’ (19).

### Public policies to address obesity

In the last 15 years, patients affected by obesity have witnessed a debate involving health, human rights, and dignity. More effective treatment options for this disorder, such as bariatric surgery and new drugs, have become available. The influence of national and international medical associations, along with the assertion of citizen's health rights and a better organization of health and legal systems, has led to greater protections and access to enhanced care. For example, the recent discussion on obesity treatments in Brazil, which was led by the National Agency of Sanitary Vigilance (ANVISA), a subordinate agency of the Ministry of Health, was a milestone in this process (20,21). Arguing for the efficacy of drugs available for treatment of obesity, the agency, based on its competence in monitoring health systems (22), permitted the debate to occur, thus increasing awareness of the obesity issue within the population. A new sociopolitical environment emerged that has led to the creation of laws that ensure access to specialized obesity care. New laws guarantee social dignity for patients with obesity, with a particular focus on their specific needs and a notable emphasis on morbid obesity, the extreme face of this disease.

### National legislation on obesity in Brazil and Argentina

In 2008, Argentina approved a national obesity law. Argentinian Federal Law No. 26.396 of 3 September 2008 (the so-called ‘Ley de Obesidad’) establishes a national policy for and approaches to the prevention of obesity (19). This law assures the creation of specific health assistance systems within general public health institutions to target obesity; it also limits the advertising of foods that contain excess fat, assures nutrition information on labels, and ensures the right to healthful food in schools. Thus, it addresses obesity as a health threat to the nation's population.

In Brazil, Article 196 of the Constitution dictates that health issues are the responsibility of local and municipal governments. Brazil took initiatives at the municipal but not at the national level. Many municipalities have joined the project ‘Lighter City’ (‘Cidade Mais Leve’) by approving laws that deal with the needs of overweight and obese patients and citizens (13).

### National jurisprudence on obesity

Compared to other legal demands, the superior courts in Brazil and Argentina still handle the obesity issue as a secondary matter. The study of judicial decisions over the past decade shows markedly opposing interpretations in the two countries. In Brazil, there is a tendency to understand obesity as a disease that deserves attention since it impairs the health of those who suffer from it; obesity is, thus, considered a type of ‘physical disability’. Conversely, in Argentina, it is regarded as a relative condition, balanced in relation to the context and the subject, taking into account factors other than obesity itself. Therefore, obesity is associated with frailty in Brazil while it is seen as a single, though not decisive, factor in judicial decisions in Argentina.

The aim of this paper is to analyze and understand the impact of obesity-related public health policies in Brazil and Argentina's legal systems. The decisions of the higher law courts of those countries are studied. Medicine, law, and public health share interests on the obesity epidemic.

## MATERIALS AND METHODS

### Diagnosis of obesity

Overweight and obesity are defined as “abnormal or excessive fat accumulation that may impair health” (2). The body mass index (BMI) is a simple index for assessing weight-for-height. According to the WHO, it is the most common measure used for classifying and defining overweight and obesity in adults. It is calculated dividing weight in kg by the square of height in metre (kg/m^2^). A BMI greater than or equal to 25 indicates overweight while one greater than or equal to 30 specifies obesity, and one of 40 or more implies severe obesity.

### Review of data

The main review was conducted using the search engines of the websites of the superior courts of Brazil and Argentina, using the key word ‘obesity’ to select the decisions in which this term was mentioned. The entire content of the juridical decisions was accessed, which means that the final decision and report, signed by the members of the courts, were analyzed and described for a better comprehension of the understanding of the legal authority on that matter. For the medical and juridical reviews of the obesity issue, searches were conducted for the academic literature on law and medicine and for online sources in Google Scholar, PubMed, Medline, juridical web pages, books, and law doctrines. The constitutions and national laws of Brazil and Argentina were examined to identify trends in their approaches to obesity. Principles of Law, such as the principle of human dignity and of the protection of law, present in both constitutions, were analyzed in the context of obesity. In Latin America today, specific legislation is almost always founded on the principles of law. The sources of data search, including constitutions, laws, and doctrines are described in Table 1.

### Obesity and litigation in law courts

Obesity is now regarded not only as a single health matter but shows also social implications. Therefore, applicants request their rights in social demands when obesity impairs their working and social capacities. The methods of this study include the analysis of these social demands, looking for trends and facts to embase and explain the reasons why health courts and other law courts have to judge increasing obesity demands on private civil rights.

### Data analysis

The content of the decisions of the ministers and courts and the specific approach of each jurist were studied in order to identify the principal methods used in resolving conflicts and addressing the issue of obesity. The decisions of judges always should rely on existing laws and reflect the principles of law and relevant specific situations. The highest courts of both countries were studied, that is, those that possess the right to interpret the laws in search of the best juridical ends.

A qualitative analysis of the votes of each magistrate was used for conceptualizing the obesity issue. The analysis involved cases dealing with (i) authorization of clinical or surgical procedures, which usually depended on given court decisions and (ii) decisions relating to the social rights of the defendants. These latter decisions are described in Table 2 and 3, which shall permit a better comprehension of the trends of each court and country towards obesity. Thus, two separate analyses for the two main groups of lawsuits were undertaken.

## RESULTS

### Recent decisions of the Supreme Court of Argentina—Corte Suprema de Justicia de la Nación (CSJN)

Ten cases involved obesity over the last 15 years.

Four were related to the treatment of disorder (three dealt with a medical error and one with the cost of bariatric surgery).

**Table 1.** Sources of data search

Source	Institution/Author	Year	Content
Argentinian Constitution	Senate of Argentina	-	Principles of Equality (Article 16), Right to Health (Article 42), and Human Rights (Article 75, item 22)
Brazilian Constitution	Senate of Brazil	1988	Principles of Human Dignity (Article 5) and the Right to Health (Article 196)
Law of Obesity	Argentine Congress	2008	Prevention of obesity; protection of those affected
Book: Judicial Politics in Argentina	Gidi A	2004	Relationship between politics and law
Article: Class Action in Brazil		2003	Civil Law
Book: Civil Law	Gaio APG	2011	Law system in Brazil
Home Page of the Supreme Court of Brazil	STF	2012	Decisions of the court, 2000-2011
Home Page of the Supreme Court of Argentina	CSJN	2012	Decisions of the court, 2000-2011

**Table 2.** ‘Social’ decisions of Argentinian Supreme Court

Date	Applicant	Defendant	Issue	Decision (For/Against the demand)	Details
09/05/2002	Obese patient	Private hospital	Moral damage	For	Had no structure to handle obese patient
26/06/2006	Individual	Newspaper	Moral damage	Against	Use of image on article about obesity
14/07/1999	Obese patient	Social Security	Retirement	Against	Obesity and hypertension–work impairment
07/04/2006	Obese Patient	Social Security	Retirement	For	Work impairment due to obesity
05/12/2000	Obese patient	Social Security	Retirement	For	Blindness associated with obesity
03/20/2003	Obese patient	Private business	Pay a debt	Against	Obesity as a reason for not to pay the debt

**Table 3.** ‘Social’ decisions of Brazilian Supreme Court

Date	Applicant	Defendant	Issue	Decision (For/Against the demand)	Details
22/03/2011	Individual	TV company	Moral damaged	Against	Use of image on article about obesity
01/03/2007	Candidate for Police	Police Dept.	Obese candidate	Against	Did not fulfil the police requirements
24/06/2004	Candidate for Air Force	Air Force	Obese candidate	Against	Did not fulfil the Air Force requirements
26/08/2009	Individual	Social Security	Recognize disability	For	Request for rent
29/06/2009	Individual	Government	Healthcare for those arrested	For	Special right for obese arrested person
19/12/2001	State of Paraná (state of Southern Brazil)	Municipalities of Paraná	Ensure that 3% of seats in culture and movie theatres are suitable for obese people	For	Right to a comfortable seat on cultural events

Six cases were referred to the social dignity of obese applicants (17). These cases deserved a specific analysis of their arguments as described in Table 2.

Table 2 shows how the Argentinian Supreme Court analyzes the obesity issue. Two cases were referred to moral damage. The first case, whose sentence was declared on 2002, regarded the use of the author's picture as an example of obesity in the national print media. The judges argued that the person's right to dignity and privacy were violated, and decided for the author's pledge.

The second case was referred to the lack of structure of the health institution to deal with the obese patient seen. This patient had an excess weight and required the special technical care that obesity demands: special beds, sphigmomanometers, specialized care; and other health apparatus which were not available at the hospital, although these were covered by the Argentinian ‘Ley de Obesidad’.

The 3 cases referring to retirement were analyzed separately according to each personal situation. One person had debts to pay and alleged to be obese but the court did not accept this explanation. In the other 2 cases, the court accepted the demand because the patients had obesity and associated morbidities which impaired their working capacity.

### Recent decisions of the Supreme Court of Brazil—Supremo Tribunal Federal (STF)

The survey found 11 obesity-related jurisprudential decisions and one monocratic decision by the president of the Supreme Court.

Six cases concerned to the costs of the treatment for the disorder. Remarkably, all but one of the latter regarded the funding of bariatric surgeries, a hot topic on treatment for obesity these days because of its shown long-term efficacy. The Brazilian court always permitted this procedure—in all 5 referred cases described in Table 3.

Six other cases concerned the right to social dignity of obese persons. Those showed different patterns, heterogeneous arguments, reasons, and facts. The decisions of the six cases relating to the social dignity of people with obesity are outlined in Table 3.

Two cases were referred to candidates to the Air Force and the Police Department. Both candidates were obese and demanded approval at the physical test, despite their condition because they sufficiently performed the logics and reasoning theory tests, having achieved high grades on those latter topics. The security forces forcefully require not only approval on theory tests but also demand specific physical conditions for acceptance because of the critical conditions of some of their job positions, such as a pilot's and a policeman's daily physical procedures. The court's decisions on both cases positioned for the special forces. The judges understood that the Principle of Equality on a contest demands the contest's bidding and purpose to be followed and should also respect the working conditions demanded from each candidate (which is different from an office work, for example).

An important case in 2001 was referred to a request of comfortable seats on cultural events at the municipality of Curitiba–Paraná (Brazil). The applicant demanded the right to an adequate seat, considering his special health situation (the usual seats were too tight, impairing his access to shows and concerts. The court decided for the applicant. The idea contained in such decision was that obese persons have the right to be treated the same way others are and may not be taken out the right to access cultural events. This was a landmark decision regarding the rights of people with excess weight, influencing other courts on similar subjects of debate.

## DISCUSSION

The Supreme Court of Argentina did not demonstrate any bias with regard to obesity in the cases studied. Their decisions varied; judges paid attention to the specific issues presented by the litigants and the peculiarities of each case. A quantitative analysis of these decisions demonstrates a ‘technical draw’, with five decisions for and five against obese patients. It is noteworthy that all cases occurred before the approval of the ‘Ley de Obesidad’ (2008), which raised public awareness of this disease and may influence future decisions of this court.

The Supreme Court of Brazil tended to favour the protection of obese individuals, regardless of the other juridical issues or facts involved in their claims. Obese people were treated as ‘victims’ of an environment and an exclusionary culture that denies them basic rights. Among the 12 cases analyzed, eight (67%) involved positive decisions for obese applicants. Furthermore, three of the four cases with unfavourable decisions were justified only by formal, constitutional and regulation factors, although the discourse that motivated and justified the rulings demonstrates the judges’ deep understanding of a fragility of those affected by the disease.

Current research and comprehension of obesity suggests that it has long ceased to be merely a matter of health and now became a major social, economic, juridical, and public policy issue in most countries of the world, especially in those who undertook the ‘nutritional transition’. The alarming growth rate of this epidemic led some western societies to think and conduct researches about the future consequences of obesity in the population. Governments, consumers, and affected populations must understand that their personal action regarding food regulation and health law discussion are essential to ensure that changes towards a healthier environment will occur.

In Brazil, the Government works on the opposite way, undertaking a public health policy towards famine called ‘Zero Hunger’ (‘Fome Zero’). This is focused on controlling famine and poverty. However, trends regarding obesity and famine have significantly changed during the last 15 years. Obese people now clearly outnumber by far those suffering from famine in a reversal of the previous trend. We believe it is time for a change in the priorities of the Federal Government of Brazil, considering the increasing obesity epidemic and the sharp decrease in famine in absolute and relative numbers.

If the current rate of increase in obesity is maintained, almost the entire population of the United States would be overweight or obese by 2048, reaching one of the most tragic pictures of the human ‘evolution’ and ‘development’.

Future generations are about to earn the consequences of inaction while the obesity rate among children and adolescents increases faster than among adults. A political, legal, and social debate is inevitable in countries that face high overweight rates. Some possibilities of solutions have already arisen in different places. One of them is by overtaxing unhealthy foods. Taxing unhealthy foods, particularly sugar-sweetened beverages, could have an important impact on the control of this epidemic. Recent evidence associates high fructose intake levels to a higher risk of the metabolic syndrome, suggesting a pattern towards weight gain. Lowering taxes of healthy products could also be a logical next step.

It was also suggested by some landmark centres of research on obesity and health law issues that national governments should lead the effort, just as they did with tobacco control. This initiative achieved success in reducing the number of smokers and the cultural habits that improve the addiction but was preceeded by a major legal suit—the 1998 ‘Tobacco Master Settlement Agreement’, suggesting that a similar deal should be sought regarding obesity control.

Another recently-discussed proposal is a better food labelling. This could be an easy, legal and democratic way of helping consumers to make healthier choices. There is some evidence pointing to this pathway.

Research on possible public policies targeting obesity started in some countries, universities, and research centres, focusing on better school environments for children and increasing exercise habits, with limited degree of success.

### Limitations

This paper addresses decisions made by the superior courts. It could have addressed judicial decisions in all levels of jurisdiction. However, the authors decided to narrow the objectives of the research, willing to illuminate the dominant juridical positions of both countries. The essential legal questions and the major problems tend to reach the superior courts.

Obesity is a complex, multifactorial health problem whose analysis certainly involves not only juridical issues but also public health, political and social matters. So, it is probable that this article would not fully reach its purposes of understanding the links and the genesis of the disease, along with its widespread consequences.

The analysis of these policies was focused on the social dignity of the subjects, not on decisions regarding funding for medical procedures. This bias could restrain the understanding of the whole public policies towards obesity. This decision aimed to highlight a topic that should be addressed in a better way than actually is. Many medical procedures and their funding already show a degree of organization. Social rights protection still do not have the same standard. The understanding of obesity should consider a wider analysis of its health, legal and social aspects.

### Conclusions

There are increasing evidences that obesity might not be solved solely through specialized healthcare. This public health challenge requires an intense interdisciplinary debate and a broad social, political and legal response. As a positive sign of slow changes going on, this discussion is leading to the creation of new juridical forums and a huge, varied literature regarding public health, health law, and health courts on the most important universities of the world.
